# 8-Chloro-4-oxo-4*H*-chromene-3-carbaldehyde

**DOI:** 10.1107/S1600536814012483

**Published:** 2014-06-04

**Authors:** Yoshinobu Ishikawa

**Affiliations:** a School of Pharmaceutical Sciences, University of Shizuoka, 52-1 Yada, Suruga-ku, Shizuoka 422-8526, Japan

## Abstract

In the title compound, C_10_H_5_ClO_3_, a chlorinated 3-formyl­chromone derivative, all atoms are essentially coplanar (r.m.s. deviation = 0.032 Å for the non-H atoms), with the largest deviation from the least-squares plane [0.0598 (14) Å] being for a pyran-ring C atom. In the crystal, mol­ecules are linked through stacking inter­actions along the *b* axis [shortest centroid–centroid distance between the pyran and benzene rings = 3.566 (2) Å].

## Related literature   

For related structures, see: Ishikawa & Motohashi (2013 ▶); Ishikawa (2014 ▶). For the synthesis of the precursor of the title compound, see: Fumagalli *et al.* (2012 ▶). For van der Waals radii; see: Bondi (1964 ▶). For halogen bonding, see: Auffinger *et al.* (2004 ▶); Metrangolo *et al.* (2005 ▶); Wilcken *et al.* (2013 ▶); Sirimulla *et al.* (2013 ▶).
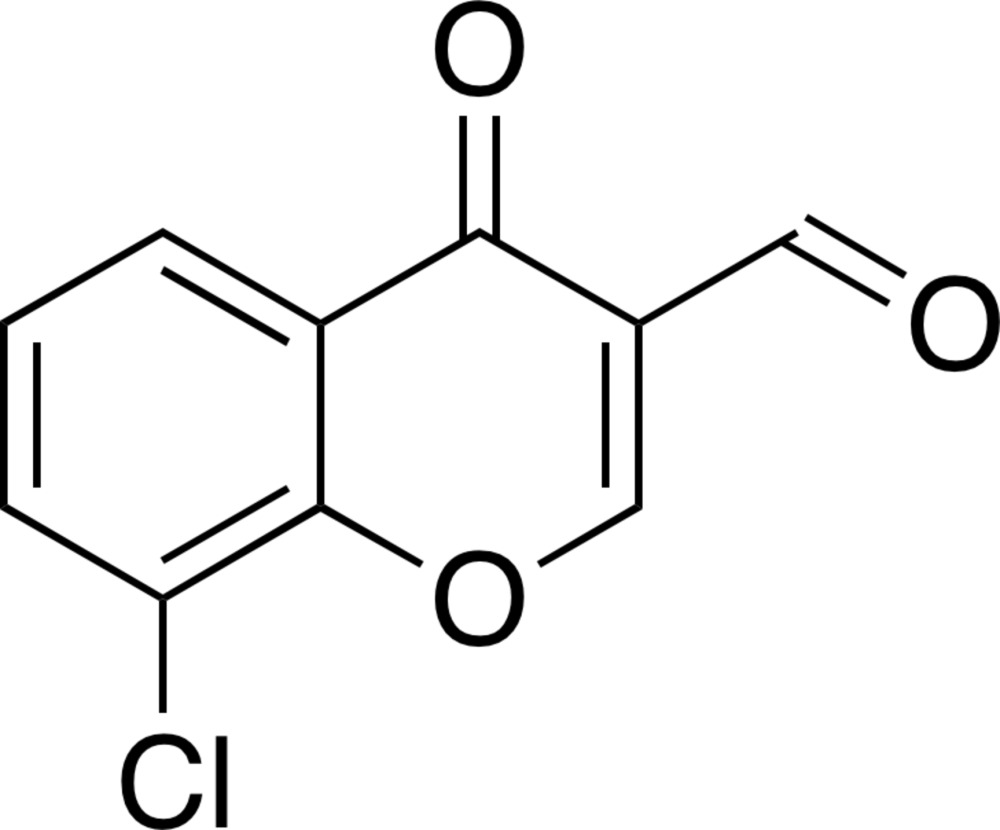


## Experimental   

### 

#### Crystal data  

C_10_H_5_ClO_3_
*M* *_r_* = 208.60
Triclinic, 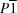

*a* = 6.9436 (15) Å
*b* = 7.1539 (17) Å
*c* = 9.165 (2) Å
α = 102.049 (19)°
β = 103.403 (17)°
γ = 100.650 (19)°
*V* = 419.89 (18) Å^3^
*Z* = 2
Mo *K*α radiation
μ = 0.43 mm^−1^
*T* = 100 K
0.38 × 0.25 × 0.10 mm

#### Data collection  

Rigaku AFC-7R diffractometer
Absorption correction: ψ scan (North *et al.*, 1968 ▶) *T*_min_ = 0.902, *T*_max_ = 0.958
2376 measured reflections
1932 independent reflections
1750 reflections with *F* ^2^ > 2σ(*F* ^2^)
*R*_int_ = 0.011
3 standard reflections every 150 reflections intensity decay: −0.039%

#### Refinement  

*R*[*F* ^2^ > 2σ(*F* ^2^)] = 0.028
*wR*(*F* ^2^) = 0.075
*S* = 1.09
1932 reflections
127 parameters
H-atom parameters constrained
Δρ_max_ = 0.34 e Å^−3^
Δρ_min_ = −0.26 e Å^−3^

### 

Data collection: *WinAFC Diffractometer Control Software* (Rigaku, 1999 ▶); cell refinement: *WinAFC Diffractometer Control Software*; data reduction: *WinAFC Diffractometer Control Software*; program(s) used to solve structure: *SIR2008* (Burla *et al.*, 2007 ▶); program(s) used to refine structure: *SHELXL97* (Sheldrick, 2008 ▶); molecular graphics: *CrystalStructure* (Rigaku, 2010 ▶); software used to prepare material for publication: *CrystalStructure*.

## Supplementary Material

Crystal structure: contains datablock(s) General, I. DOI: 10.1107/S1600536814012483/zl2591sup1.cif

Structure factors: contains datablock(s) I. DOI: 10.1107/S1600536814012483/zl2591Isup2.hkl

Supporting information file. DOI: 10.1107/S1600536814012483/zl2591Isup3.cml

Additional supporting information:  crystallographic information; 3D view; checkCIF report

## Figures and Tables

**Fig. 1.**
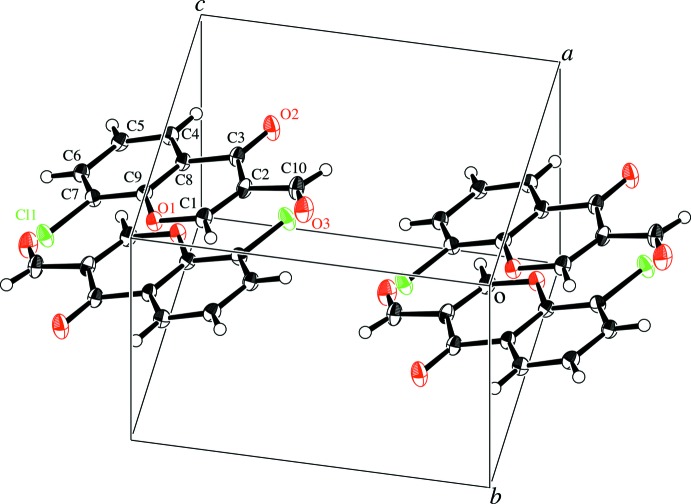
A packing view of the title compound, with displacement ellipsoids drawn at the 50% probability level. Hydrogen atoms are shown as small spheres of arbitrary radius.

**Fig. 2.**
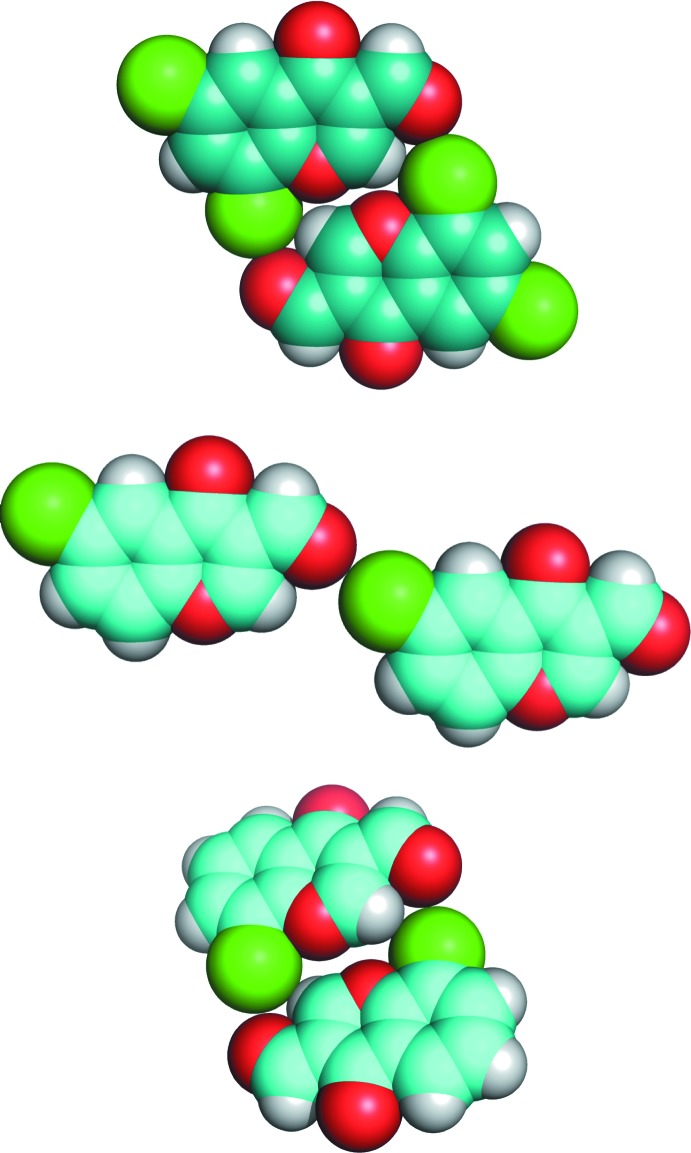
Sphere models of the crystal structures of 6,8-dichloro-4-oxochromene-3-carbaldehyde (top), 6-chloro-4-oxo-4H-chromene-3-carbaldehyde (middle), and the title compound (bottom).

## supplementary crystallographic information

### S1. Comment

Halogen bonds have been found to occur in organic, inorganic, and biological systems, and have recently attracted much attention in medicinal chemistry, chemical biology and supramolecular chemistry (Auffinger *et al.*, 2004, Metrangolo *et al.*, 2005, Wilcken *et al.*, 2013, Sirimulla *et al.*, 2013). We have recently reported the crystal structures of chlorinated 3-formylchromone derivatives 6,8-dichloro-4-oxochromene-3-carbaldehyde (Ishikawa & Motohashi, 2013, Fig.2 (top)) and 6-chloro-4-oxo-4*H*-chromene-3-carbaldehyde (Ishikawa, 2014, Fig.2 (middle)). It was found that a halogen bond is formed for 6,8-dichloro-4-oxochromene-3-carbaldehyde between the formyl oxygen atom and the chlorine atom at the 8-position, but none is formed for 6-chloro-4-oxo-4*H*-chromene-3-carbaldehyde between the formyl oxygen atom and the chlorine atom at the 6-position. As part of our interest in this type of chemical bonding, we herein report the crystal structure of a monochlorinated 3-formylchromone derivative 8-chloro-4-oxo-4*H*-chromene-3-carbaldehyde. The objective of this study is to reveal whether halogen bond(*s*) can be formed in the crystal of the title compound with the chlorine atom at 8-position and without a halogen atom at 6-position.

The mean deviation of the least-square planes for the non-hydrogen atoms is 0.0316 Å, and the largest deviation is 0.0598 (14) Å for C1. These mean that these atoms are essentially coplanar. In the crystal, the molecules are stacked with their inversion-symmetry equivalent along the *b*-axis direction [centroid–centroid distance between the pyran and benzene rings of the 4*H*-chromene units = 3.566 (2) Å, symmetry operator i: -*x* + 1, -*y* + 1, -*z* + 2], as shown in Fig.1.

The distance between the chlorine atom and the formyl oxygen atom of the translation-symmetry equivalent [Cl1···O3^ii^ = 3.301 (2) Å, ii: *x*, *y*, *z* + 2] is nearly equal to the sum of their van der Waals radii [3.27 Å] (Bondi, 1964), as shown at the bottom of Fig.2. Thus, it is concluded that there is no halogen bond in the title compound. On the other hand, the angles of C–Cl···O (157.15 (6)°) and Cl···O=C (129.24 (10)°) are close to those of 6,8-dichloro-4-oxochromene-3-carbaldehyde, (C–Cl···O (160.4 (3)°) and Cl···O=C (138.7 (4)°), Fig.2(top)). Thus, the significance of the vicinal electron-withdrawing substituent in forming of a halogen bond (Wilcken *et al.*, 2013) is crystallographically validated from the fact that halogen bonding is observed in the dichlorinated 3-formylchromone, but is not observed in the monochlorinated ones. These results should be invaluable for rational drug design.

### S2. Experimental

2-Hydroxy-3-chloroacetophenone was prepared according to a literature method (Fumagalli *et al.*, 2012). To a solution of 2-hydroxy-3-chloroacetophenone (11.1 mmol) in *N*,*N*-dimethylformamide (30 ml) was added dropwise POCl_3_ (27.7 mmol) for 5 min at 0 °C. After the mixture was stirred for 16 h at room temperature, water (50 ml) was added. The precipitates were collected, washed with water, and dried *in vacuo* (yield: 72%). ^1^H NMR (400 MHz, DMSO-*d*_6_): *δ* = 7.58 (t, 1H, *J* = 7.8 Hz), 8.07 (d, 1H, *J* = 7.8 Hz), 8.10 (d, 1H, *J* = 7.8 Hz), 9.03 (s, 1H), 10.12 (s, 1H). DART-MS calcd for [C_10_H_5_Cl_1_O_3_ + H^+^]: 209.001, found 209.014. Single crystals suitable for X-ray diffraction were obtained by slow evaporation of a chloroform solution of the title compound at room temperature.

### S3. Refinement

The C(*sp*^2^)-bound hydrogen atoms were placed in geometrical positions [C–H 0.95 Å, *U*_iso_(H) = 1.2*U*_eq_(C)], and refined using a riding model.

### Crystal data

**Table:**

C_10_H_5_ClO_3_	*Z* = 2
*M**_r_* = 208.60	*F*(000) = 212.00
Triclinic, *P*1	*D*_x_ = 1.650 Mg m^−^^3^
Hall symbol: -P 1	Mo *K*α radiation, λ = 0.71069 Å
*a* = 6.9436 (15) Å	Cell parameters from 25 reflections
*b* = 7.1539 (17) Å	θ = 15.1–17.5°
*c* = 9.165 (2) Å	µ = 0.43 mm^−^^1^
α = 102.049 (19)°	*T* = 100 K
β = 103.403 (17)°	Plate, yellow
γ = 100.650 (19)°	0.38 × 0.25 × 0.10 mm
*V* = 419.89 (18) Å^3^	

### Data collection

**Table:**

Rigaku AFC-7R diffractometer	*R*_int_ = 0.011
ω–2θ scans	θ_max_ = 27.5°
Absorption correction: ψ scan (North *et al.*, 1968)	*h* = −5→9
*T*_min_ = 0.902, *T*_max_ = 0.958	*k* = −9→9
2376 measured reflections	*l* = −11→11
1932 independent reflections	3 standard reflections every 150 reflections
1750 reflections with *F*^2^ > 2σ(*F*^2^)	intensity decay: −0.039%

### Refinement

**Table:**

Refinement on *F*^2^	Secondary atom site location: difference Fourier map
*R*[*F*^2^ > 2σ(*F*^2^)] = 0.028	Hydrogen site location: inferred from neighbouring sites
*wR*(*F*^2^) = 0.075	H-atom parameters constrained
*S* = 1.09	*w* = 1/[σ^2^(*F*_o_^2^) + (0.0344*P*)^2^ + 0.1939*P*] where *P* = (*F*_o_^2^ + 2*F*_c_^2^)/3
1932 reflections	(Δ/σ)_max_ < 0.001
127 parameters	Δρ_max_ = 0.34 e Å^−^^3^
0 restraints	Δρ_min_ = −0.26 e Å^−^^3^
Primary atom site location: structure-invariant direct methods	

### Special details

**Table:**

Refinement. Refinement was performed using all reflections. The weighted *R*-factor (*wR*) and goodness of fit (*S*) are based on *F*^2^. *R*-factor (gt) are based on *F*. The threshold expression of *F*^2^ > 2.0 σ(*F*^2^) is used only for calculating *R*-factor (gt).

### Fractional atomic coordinates and isotropic or equivalent isotropic displacement parameters (Å^2^)

**Table:**

	*x*	*y*	*z*	*U*_iso_*/*U*_eq_	
Cl1	0.28721 (5)	0.37563 (5)	1.29528 (4)	0.02580 (11)	
O1	0.21886 (13)	0.15266 (13)	0.97744 (10)	0.0187 (2)	
O2	0.63950 (15)	0.10011 (16)	0.73295 (11)	0.0279 (3)	
O3	0.04173 (16)	−0.20711 (16)	0.52591 (12)	0.0297 (3)	
C1	0.16551 (19)	0.04262 (19)	0.82917 (14)	0.0189 (3)	
C2	0.29522 (19)	0.01959 (19)	0.74169 (14)	0.0184 (3)	
C3	0.51287 (19)	0.11867 (19)	0.80487 (14)	0.0184 (3)	
C4	0.76947 (18)	0.35541 (19)	1.04110 (15)	0.0180 (3)	
C5	0.81834 (19)	0.47543 (19)	1.19027 (15)	0.0189 (3)	
C6	0.66911 (19)	0.48195 (18)	1.26931 (14)	0.0183 (3)	
C7	0.47153 (19)	0.37036 (19)	1.19772 (14)	0.0176 (3)	
C8	0.56861 (18)	0.24423 (18)	0.96597 (14)	0.0161 (3)	
C9	0.41997 (18)	0.25375 (17)	1.04458 (14)	0.0157 (3)	
C10	0.2152 (2)	−0.1091 (2)	0.58050 (15)	0.0236 (3)	
H1	0.0257	−0.0238	0.7828	0.0227*	
H2	0.8725	0.3481	0.9891	0.0216*	
H3	0.3054	−0.1145	0.5169	0.0284*	
H4	0.9536	0.5537	1.2392	0.0226*	
H5	0.7035	0.5632	1.3724	0.0220*	

### Atomic displacement parameters (Å^2^)

**Table:**

	*U*^11^	*U*^22^	*U*^33^	*U*^12^	*U*^13^	*U*^23^
Cl1	0.01652 (16)	0.0338 (2)	0.02036 (17)	−0.00018 (12)	0.00735 (12)	−0.00404 (13)
O1	0.0116 (4)	0.0215 (5)	0.0159 (5)	−0.0032 (4)	0.0015 (4)	−0.0009 (4)
O2	0.0227 (5)	0.0370 (6)	0.0190 (5)	−0.0015 (5)	0.0093 (4)	0.0010 (4)
O3	0.0252 (6)	0.0309 (6)	0.0200 (5)	−0.0059 (5)	0.0003 (4)	−0.0027 (4)
C1	0.0158 (6)	0.0182 (6)	0.0159 (6)	−0.0030 (5)	−0.0003 (5)	0.0014 (5)
C2	0.0187 (6)	0.0174 (6)	0.0138 (6)	−0.0019 (5)	0.0009 (5)	0.0026 (5)
C3	0.0182 (6)	0.0197 (6)	0.0144 (6)	−0.0004 (5)	0.0032 (5)	0.0041 (5)
C4	0.0134 (6)	0.0200 (6)	0.0183 (6)	−0.0005 (5)	0.0038 (5)	0.0047 (5)
C5	0.0132 (6)	0.0200 (6)	0.0185 (6)	−0.0012 (5)	0.0004 (5)	0.0039 (5)
C6	0.0173 (6)	0.0177 (6)	0.0147 (6)	0.0006 (5)	0.0005 (5)	0.0006 (5)
C7	0.0147 (6)	0.0196 (6)	0.0165 (6)	0.0019 (5)	0.0044 (5)	0.0026 (5)
C8	0.0147 (6)	0.0169 (6)	0.0140 (6)	−0.0002 (5)	0.0025 (5)	0.0039 (5)
C9	0.0116 (6)	0.0154 (6)	0.0157 (6)	−0.0011 (5)	0.0004 (5)	0.0026 (5)
C10	0.0248 (7)	0.0257 (7)	0.0138 (6)	−0.0017 (6)	0.0024 (5)	0.0014 (5)

### Geometric parameters (Å, º)

**Table:**

Cl1—C7	1.7243 (16)	C4—C8	1.4029 (16)
O1—C1	1.3475 (15)	C5—C6	1.397 (2)
O1—C9	1.3763 (14)	C6—C7	1.3817 (17)
O2—C3	1.2250 (19)	C7—C9	1.4006 (17)
O3—C10	1.2061 (16)	C8—C9	1.393 (2)
C1—C2	1.347 (2)	C1—H1	0.950
C2—C3	1.4658 (17)	C4—H2	0.950
C2—C10	1.4836 (17)	C5—H4	0.950
C3—C8	1.4797 (17)	C6—H5	0.950
C4—C5	1.3815 (18)	C10—H3	0.950
			
Cl1···O1	2.8973 (12)	C3···H1	3.2929
O1···C3	2.8719 (19)	C3···H2	2.6746
O2···C1	3.574 (2)	C3···H3	2.7084
O2···C4	2.8604 (17)	C4···H5	3.2636
O2···C10	2.9089 (18)	C6···H2	3.2648
O3···C1	2.8120 (17)	C7···H4	3.2634
C1···C7	3.5981 (19)	C8···H4	3.2730
C1···C8	2.7591 (18)	C9···H1	3.1860
C2···C9	2.7695 (18)	C9···H2	3.2689
C4···C7	2.783 (2)	C9···H5	3.2672
C5···C9	2.7806 (18)	C10···H1	2.5482
C6···C8	2.7921 (18)	H1···H3	3.4825
Cl1···O2^i^	3.4989 (15)	H2···H4	2.3282
Cl1···O3^ii^	3.3012 (15)	H4···H5	2.3459
Cl1···C5^iii^	3.4247 (16)	Cl1···H1^ii^	2.8415
O1···O1^ii^	3.5617 (16)	Cl1···H2^iii^	3.4669
O1···O2^i^	3.5683 (17)	Cl1···H4^iii^	2.8395
O1···C3^i^	3.5282 (19)	Cl1···H5^x^	2.9688
O1···C4^iv^	3.5456 (19)	O1···H1^ii^	3.2499
O1···C5^iv^	3.359 (2)	O1···H2^iii^	3.0086
O1···C8^i^	3.5096 (19)	O1···H4^iv^	3.3704
O2···Cl1^i^	3.4989 (15)	O2···H1^viii^	2.9439
O2···O1^i^	3.5683 (17)	O2···H3^v^	2.4269
O2···C7^i^	3.534 (2)	O2···H4^xi^	3.3161
O2···C9^i^	3.591 (2)	O3···H1^vi^	3.5460
O2···C10^v^	3.267 (2)	O3···H4^vii^	2.6830
O3···Cl1^ii^	3.3012 (15)	O3···H5^vii^	2.5041
O3···O3^vi^	3.2307 (19)	O3···H5^i^	3.5184
O3···C5^vii^	3.2551 (18)	C1···H2^iii^	3.5714
O3···C6^vii^	3.1687 (17)	C1···H2^i^	3.5400
O3···C6^i^	3.560 (2)	C1···H4^iv^	3.2889
O3···C10^vi^	3.295 (2)	C2···H5^iv^	3.3614
C1···C4^i^	3.371 (3)	C3···H3^v^	3.4629
C1···C5^iv^	3.472 (3)	C3···H5^iv^	3.4282
C1···C8^i^	3.542 (3)	C4···H1^i^	3.4694
C2···C6^iv^	3.553 (3)	C4···H2^xi^	3.0614
C2···C7^i^	3.552 (3)	C5···H2^xi^	3.2259
C2···C9^i^	3.578 (2)	C6···H3^i^	3.5976
C3···O1^i^	3.5282 (19)	C10···H4^vii^	3.3912
C3···C6^iv^	3.460 (3)	C10···H5^i^	3.4945
C3···C7^i^	3.515 (3)	H1···Cl1^ii^	2.8415
C3···C9^i^	3.304 (3)	H1···O1^ii^	3.2499
C4···O1^iv^	3.5456 (19)	H1···O2^iii^	2.9439
C4···C1^i^	3.371 (3)	H1···O3^vi^	3.5460
C4···C7^iv^	3.570 (3)	H1···C4^i^	3.4694
C4···C9^iv^	3.459 (3)	H1···H2^iii^	3.4159
C5···Cl1^viii^	3.4247 (16)	H1···H2^i^	3.4928
C5···O1^iv^	3.359 (2)	H1···H3^vi^	3.5853
C5···O3^ix^	3.2551 (18)	H1···H4^iv^	3.3899
C5···C1^iv^	3.472 (3)	H2···Cl1^viii^	3.4669
C5···C9^iv^	3.521 (2)	H2···O1^viii^	3.0086
C6···O3^ix^	3.1687 (17)	H2···C1^viii^	3.5714
C6···O3^i^	3.560 (2)	H2···C1^i^	3.5400
C6···C2^iv^	3.553 (3)	H2···C4^xi^	3.0614
C6···C3^iv^	3.460 (3)	H2···C5^xi^	3.2259
C6···C8^iv^	3.538 (2)	H2···H1^viii^	3.4159
C6···C10^i^	3.387 (3)	H2···H1^i^	3.4928
C7···O2^i^	3.534 (2)	H2···H2^xi^	2.4762
C7···C2^i^	3.552 (3)	H2···H4^xi^	2.7931
C7···C3^i^	3.515 (3)	H3···O2^v^	2.4269
C7···C4^iv^	3.570 (3)	H3···C3^v^	3.4629
C7···C8^iv^	3.427 (3)	H3···C6^i^	3.5976
C8···O1^i^	3.5096 (19)	H3···H1^vi^	3.5853
C8···C1^i^	3.542 (3)	H3···H3^v^	3.0081
C8···C6^iv^	3.538 (2)	H3···H4^vii^	3.2450
C8···C7^iv^	3.427 (3)	H3···H5^i^	3.5572
C8···C9^i^	3.560 (2)	H4···Cl1^viii^	2.8395
C8···C9^iv^	3.600 (2)	H4···O1^iv^	3.3704
C9···O2^i^	3.591 (2)	H4···O2^xi^	3.3161
C9···C2^i^	3.578 (2)	H4···O3^ix^	2.6830
C9···C3^i^	3.304 (3)	H4···C1^iv^	3.2889
C9···C4^iv^	3.459 (3)	H4···C10^ix^	3.3912
C9···C5^iv^	3.521 (2)	H4···H1^iv^	3.3899
C9···C8^i^	3.560 (2)	H4···H2^xi^	2.7931
C9···C8^iv^	3.600 (2)	H4···H3^ix^	3.2450
C10···O2^v^	3.267 (2)	H5···Cl1^x^	2.9688
C10···O3^vi^	3.295 (2)	H5···O3^ix^	2.5041
C10···C6^i^	3.387 (3)	H5···O3^i^	3.5184
Cl1···H5	2.8072	H5···C2^iv^	3.3614
O2···H2	2.5915	H5···C3^iv^	3.4282
O2···H3	2.6355	H5···C10^i^	3.4945
O3···H1	2.4818	H5···H3^i^	3.5572
C1···H3	3.2782		
			
C1—O1—C9	118.02 (11)	C4—C8—C9	119.19 (11)
O1—C1—C2	125.01 (11)	O1—C9—C7	117.14 (12)
C1—C2—C3	120.64 (11)	O1—C9—C8	122.57 (10)
C1—C2—C10	119.09 (11)	C7—C9—C8	120.29 (11)
C3—C2—C10	120.26 (13)	O3—C10—C2	123.51 (15)
O2—C3—C2	123.90 (11)	O1—C1—H1	117.494
O2—C3—C8	122.20 (11)	C2—C1—H1	117.497
C2—C3—C8	113.90 (12)	C5—C4—H2	119.817
C5—C4—C8	120.37 (13)	C8—C4—H2	119.815
C4—C5—C6	120.11 (11)	C4—C5—H4	119.941
C5—C6—C7	120.09 (11)	C6—C5—H4	119.944
Cl1—C7—C6	120.35 (10)	C5—C6—H5	119.956
Cl1—C7—C9	119.75 (10)	C7—C6—H5	119.956
C6—C7—C9	119.90 (13)	O3—C10—H3	118.246
C3—C8—C4	121.00 (13)	C2—C10—H3	118.242
C3—C8—C9	119.81 (10)		
			
C1—O1—C9—C7	178.77 (11)	C8—C4—C5—C6	2.1 (3)
C1—O1—C9—C8	−0.80 (18)	C8—C4—C5—H4	−177.9
C9—O1—C1—C2	1.7 (2)	H2—C4—C5—C6	−177.9
C9—O1—C1—H1	−178.3	H2—C4—C5—H4	2.1
O1—C1—C2—C3	−0.5 (3)	H2—C4—C8—C3	−1.5
O1—C1—C2—C10	179.02 (12)	H2—C4—C8—C9	178.9
H1—C1—C2—C3	179.5	C4—C5—C6—C7	−0.9 (2)
H1—C1—C2—C10	−1.0	C4—C5—C6—H5	179.1
C1—C2—C3—O2	178.13 (14)	H4—C5—C6—C7	179.1
C1—C2—C3—C8	−1.5 (2)	H4—C5—C6—H5	−0.9
C1—C2—C10—O3	−5.7 (3)	C5—C6—C7—Cl1	179.30 (12)
C1—C2—C10—H3	174.3	C5—C6—C7—C9	−1.3 (2)
C3—C2—C10—O3	173.77 (13)	H5—C6—C7—Cl1	−0.7
C3—C2—C10—H3	−6.2	H5—C6—C7—C9	178.7
C10—C2—C3—O2	−1.3 (3)	Cl1—C7—C9—O1	2.11 (18)
C10—C2—C3—C8	179.06 (12)	Cl1—C7—C9—C8	−178.31 (9)
O2—C3—C8—C4	3.0 (3)	C6—C7—C9—O1	−177.27 (12)
O2—C3—C8—C9	−177.39 (13)	C6—C7—C9—C8	2.3 (2)
C2—C3—C8—C4	−177.42 (12)	C3—C8—C9—O1	−1.2 (2)
C2—C3—C8—C9	2.21 (19)	C3—C8—C9—C7	179.27 (11)
C5—C4—C8—C3	178.54 (12)	C4—C8—C9—O1	178.46 (12)
C5—C4—C8—C9	−1.1 (2)	C4—C8—C9—C7	−1.1 (2)

Symmetry codes: (i) −*x*+1, −*y*, −*z*+2; (ii) −*x*, −*y*, −*z*+2; (iii) *x*−1, *y*, *z*; (iv) −*x*+1, −*y*+1, −*z*+2; (v) −*x*+1, −*y*, −*z*+1; (vi) −*x*, −*y*, −*z*+1; (vii) *x*−1, *y*−1, *z*−1; (viii) *x*+1, *y*, *z*; (ix) *x*+1, *y*+1, *z*+1; (x) −*x*+1, −*y*+1, −*z*+3; (xi) −*x*+2, −*y*+1, −*z*+2.
